# Assessing Night-to-Night Sleep Variability as a Hallmark of Chronic Insomnia Using Longitudinal, Contactless, Mobile Sleep Monitoring: Prospective Cohort Study

**DOI:** 10.2196/73969

**Published:** 2026-03-18

**Authors:** Devon A Hansen, Mary E Peterson, Myles G Finlay, Elie Gottlieb, Sharon Danoff-Burg, Roy JEM Raymann, Dedra Buchwald, Nathaniel F Watson

**Affiliations:** 1 Department of Translational Medicine and Physiology, Sleep and Performance Research Center, Elson S. Floyd College of Medicine, Washington State University, 412 E. Spokane Falls Blvd., Spokane, WA, 99202, United States, 1 5093587750; 2 Division of Behavioral and Organizational Sciences, Claremont Graduate University, Claremont, CA, United States; 3 Sleep.ai, Carlsbad, CA, United States; 4 Department of Neurological Surgery, University of Washington, Seattle, WA, United States; 5 Department of Neurology, University of Washington Medicine Sleep Center, University of Washington School of Medicine, University of Washington, Seattle, WA, United States

**Keywords:** insomnia, consumer sleep technology, noncontact sleep monitoring, insomnia phenotype, night-to-night sleep variability, sleep efficiency, sleep latency, intermittent wakefulness

## Abstract

**Background:**

Chronic insomnia affects more than 30% of US adults, is more prevalent in women and older adults, and is strongly associated with poor mental and physical health outcomes. Poor sleep quality and intraindividual variability of sleep are recognized to be key characteristics of chronic insomnia, but longitudinal assessment of sleep is largely subjective, with no objective characterization of sleep patterns and intraindividual variability over extended periods. Objective, ecologically valid longitudinal sleep measurements are needed to help identify and manage insomnia in both clinical and population settings. Consumer sleep technologies offer a possible solution, but their clinical utility remains relatively unexplored.

**Objective:**

We aimed to evaluate the utility of a contactless, radio frequency–based device by demonstrating its ability to objectively characterize sleep in individuals with insomnia over an extended observation period in a naturalistic home environment.

**Methods:**

Eighty-three participants meeting criteria for chronic insomnia and 29 healthy good-sleeper controls underwent 8 consecutive weeks of home-based sleep monitoring using an objective, contactless, radio frequency–based sleep monitoring device. Sleep efficiency, sleep latency, intermittent wakefulness, time in bed, and total sleep time were objectively quantified as daily means and SDs.

**Results:**

Compared to healthy controls, individuals with chronic insomnia exhibited reduced sleep efficiency, increased sleep latency, and increased intermittent wakefulness. They also demonstrated significantly higher night-to-night variability (SDs) in sleep efficiency, sleep latency, and intermittent wakefulness than good-sleeper controls (all *P*<.001).

**Conclusions:**

In the longest known objective characterization of sleep among individuals with chronic insomnia, we show that a radio frequency–based, contactless sleep monitoring device deployed in the participants’ typical sleep environments accurately distinguished healthy good sleepers from those with insomnia. Importantly, we show that persistent night-to-night variability in objective sleep measures is a hallmark of chronic insomnia.

## Introduction

Chronic insomnia is a significant public health concern, affecting more than 30% of US adults [1], with similarly high rates of persistence at 1 year [2]. Insomnia is more prevalent in women and middle-aged to older adults and is strongly associated with poor mental and physical health outcomes [2]. Insomnia involves difficulty initiating or maintaining sleep or early morning awakenings occurring at least 3 nights per week for at least 3 months. According to the *International Classification of Sleep Disorders*, impairment in daytime functioning must also be present [3]. Poor sleep quality and intraindividual variability of sleep over time are considered key characteristics of chronic insomnia [4-7]. Specific sleep concerns and comorbidities vary among individuals with chronic insomnia, making evaluation and management challenging [8]. Presently, longitudinal assessment of sleep in people with insomnia is largely subjective, with no objective characterization of sleep patterns and intraindividual variability over extended periods. Objective, ecologically valid longitudinal sleep measurements are needed to help identify and manage insomnia at clinical and population levels.

Assessment of sleep using polysomnography (PSG) is not indicated for insomnia due to reverse first-night effects, in which sleep paradoxically improves in the laboratory setting [9]. Wrist actigraphy has been used to assess sleep in naturalistic settings [10-16]. These data, typically averaged within individuals over time, generally conclude limited differences between patients with insomnia and healthy controls, which leads researchers and practitioners to conclude that objective sleep differences between individuals with insomnia and healthy, good-sleeper controls are generally limited and minor [6,7,17,18]. However, this approach overlooks the marked intraindividual variability observed in some individuals with chronic insomnia [8,19]. Intraindividual variability in sleep parameters correlates with self-reported ratings of sleep quality, suggesting that the most challenging aspect of insomnia may not be continual exposure to poor sleep but rather night-to-night inconsistency of sleep [17].

For the benefit of both research and clinical practice, more extensive, objective, longitudinal, ecologically valid research is needed regarding the naturalistic sleep of individuals with chronic insomnia. This is modestly achievable with wrist actigraphy [20-22]; however, the measurement duration is substantially limited by battery life to approximately 2 weeks of monitoring. Furthermore, users must remember to wear the actigraph during sleep periods, and depending on the actigraph model, user, researcher, or practitioner, interaction may be required to download the data. Wearing the actigraph during sleep can be intrusive and thus negatively influence the very sleep it is measuring. Actigraphic data also require further processing [23] and scoring to reliably assess sleep parameters day by day. Finally, recent performance evaluations of traditional actigraphy highlight its poor specificity (ie, wake detection) relative to more novel and emerging consumer sleep technology (CST) devices [22]. The underestimation of wake after sleep onset—commonly observed with actigraphy measurement—is particularly problematic in populations with sleep disorders such as insomnia, in which sleep fragmentation is a typical sequela.

New CSTs involving contactless sleep measurement from the bedside may provide a solution to overcome these limitations. One such bedside sleep monitoring device is the SleepScore Max (SSM; SleepScore Labs). This noncontact device uses ultra–low energy radar to track bodily movements and respiration patterns through measurement of chest cavity motion to determine sleep stages. The SSM also measures light levels and ambient room temperature. The device is wall-powered by an AC adapter and communicates with a compatible smartphone through Bluetooth technology. If the Bluetooth connection is lost or interrupted, the SSM continues to track sleep as long as power is maintained [24]. Recorded data are transferred from the SSM to the smartphone for processing to identify the presence of a sleeper and to determine sleep periods and stages. The user is required to start and stop a recording in an associated app, after which the processed results are uploaded from the smartphone to the cloud. The contactless nature of the SSM may also lessen the chance of the measurement itself impacting sleep. Researchers affiliated with academic or clinical institutions can access the data stored in the cloud through a download portal provided by SleepScore Labs.

The SSM has been validated against polysomnography and wrist actigraphy in healthy sleepers [22], while previous generations of the device (eg, S+ and SleepMinder) have been validated in both healthy sleepers [25,26] and in patients with obstructive sleep apnea (OSA) [27,28]. Relative to polysomnography in healthy sleepers, the SSM’s sensitivity to detect sleep was 94%, with a specificity of 50% [22]. For comparison, the sensitivity of wrist actigraphy is as high as 97%, but its specificity is lower at 39% [22]. In OSA, the SleepMinder’s sensitivity to detect sleep is 86%, with a specificity of 52%. In a head-to-head comparison using the SleepMinder in patients with OSA, actigraphy showed slightly better sensitivity at 94% but substantially lower specificity at 34% [28]. Thus, the SSM’s ability to monitor sleep naturalistically is comparable to wrist actigraphy, with the addition of long-term sleep monitoring, a contactless design, and higher specificity. This makes CSTs such as the SSM more suitable for long-duration studies of the naturalistic sleep of individuals with chronic insomnia.

Here, we present the results from the longest known objective assessment of sleep in people with chronic insomnia. Both groups underwent 8 consecutive weeks of contactless, naturalistic sleep monitoring using the SSM. On the basis of this study design, we compared daily means and daily variabilities of sleep between individuals with chronic insomnia and good-sleeper controls. Our objective was to determine the utility of a contactless, radio frequency–based device and to demonstrate its ability to characterize sleep in individuals with insomnia over an extended period of observation in a naturalistic environment.

## Methods

### Overview

Data presented here are part of a larger clinical trial to examine the efficacy of the SSM to (1) accurately measure naturalistic sleep at home in people with chronic insomnia as compared to healthy-sleeper controls and wrist-worn actigraphy and (2) test the ability of the SSM’s integrated sleep coaching program to improve sleep in people with chronic insomnia compared to an active control group using the current standard of care, online cognitive behavioral therapy for insomnia, and passive control groups of individuals with chronic insomnia and healthy-sleeper controls. All participants collected 8 weeks of continuous nightly recordings using the SSM, with 1 week of wrist actigraphy recordings preceding and 1 week following the 8-week monitoring phase. We planned to enroll 90 carefully screened adults with chronic insomnia and 30 carefully screened healthy adult controls. The chronic insomnia group was randomized to 1 of 3 groups: SSM alone (passive control), SSM with online cognitive behavioral therapy for insomnia (active control), and SSM with a coaching program (test intervention), relative to healthy controls using the SSM alone. Here, we present results from aim 1, which was to evaluate the ability of the SSM to accurately measure sleep at home in people with chronic insomnia as compared to healthy-sleeper controls.

### Power Analysis

Our preliminary data from a prior study with 17 chronic sleep-onset insomniacs and 29 healthy controls show a large effect size (Cohen d=1.99) for between-group differences. Using this effect size in our power calculations, we determined that 30 participants must be enrolled per group (90 total) in the proposed study to achieve 80% statistical power to address each of our aims at a type I error threshold of .05.

### Participants

We enrolled adults with chronic insomnia as well as healthy-sleeper controls. Participants were recruited via print and online advertisements. Recruitment took place between November 2019 and November 2021. Six participants were screened in the laboratory and completed data collection before the COVID-19 pandemic. All other participants were screened remotely—through videoconference meetings and secure administration of online questionnaires—to adhere to COVID-19 social distancing requirements.

Prior to the start of the at-home sleep monitoring study, participants were screened via an interview with trained staff to determine whether they met the eligibility criteria. All participants were aged 18 to 65 years. Participants in the chronic insomnia group met criteria for chronic insomnia as defined by the *International Classification of Sleep Disorders*, 3rd edition, with no other clinically relevant condition contributing to their reported sleep disturbance [3]. Specifically, individuals with insomnia had difficulty sleeping at least 3 times per week for a period of at least 3 months. In addition, those with insomnia reported having at least 1 of the following next-day consequences: fatigue or malaise; attention, concentration, or memory impairment; impaired social, family, occupational, or academic performance; mood disturbances or irritability; daytime sleepiness; behavioral problems (eg, hyperactivity, impulsivity, and aggression); reduced motivation, energy, or initiative; proneness to errors or accidents; or concerns about or dissatisfaction with sleep. They had no other diagnosed sleep disorder other than chronic insomnia, as assessed by questionnaire. The reported sleep or wake concerns could not be explained by inadequate opportunity (ie, enough time allotted for sleep) or inadequate circumstances (ie, an environment that was safe, dark, quiet, and comfortable) for sleep.

Individuals in the healthy good-sleeper control group reported no current clinically relevant history of medical disorders or other illnesses and were free of suspected sleep disorders, as determined by the STOP-BANG (snoring, tiredness, observed apnea, pressure [high blood pressure], BMI, age, neck circumference, and gender) questionnaire [29] (score no greater than low risk for OSA) and the Pittsburgh Sleep Quality Index (score<5) [30]. Participants were required to have daily access to an iPhone (Apple Inc) to run the smartphone app associated with the SSM. All participants reported that they were not currently engaged in shift work.

### Ethical Considerations

All participants provided written informed consent, and the study was approved by the institutional review board of Washington State University (protocol 17379). All sleep recording data were collected via participant IDs rather than direct identifiers. Upon completion of the study, participants were compensated with a US $20 gift card and were allowed to keep their SSM device.

### Procedure

For data collection, participants were instructed to place the SSM within arm’s length on their bedside table or nightstand and to collect 8 consecutive weeks of nightly sleep recordings. Participants manually initiated and ended each nightly recording through the smartphone app associated with the SSM.

### Instrument

The SSM recorded sleep data in 30-second epochs classified as “wake,” “light,” “deep,” “rapid eye movement,” and “absence.” “Absence” denotes that the subject was not present in the bed. The analysis window begins at the first epoch following the last absence preceding sleep onset and ends at the first absence after the final sleep epoch or at the end of the recording, whichever occurs first. Data were measured by the SSM and processed by the associated smartphone app. Time in bed and sleep parameters were estimated based on ultra-low energy radar measurements and were automatically uploaded to the cloud and later downloaded by the researchers. These data were linked to and coded for each participant using a unique study ID.

The following SSM sleep parameters were assessed for each of the recorded nights:

- Adjusted sleep latency (SL_ad__j_)—if absence occurs before the first sleep epoch, SL_adj_ = t_first sleep_ – t_last Absence before sleep_; otherwise SL_adj_ = sleep latency
- Total sleep time (TST)—sum of light, deep, and rapid eye movement epochs within the analysis window
- Wake after sleep onset (WASO)—wake time *after* sleep onset and *before* final awakening; absence epochs were excluded
- Final awakening—time from the last sleep epoch to the end boundary (first absence after sleep or recording end)
- Time in bed—presence-based time in bed
- Sleep efficiency—ratio of TST to time in bed
- Rounding—analyses use epoch totals (0.5-minute granularity). The app displays TST and WASO rounded to whole minutes; mixing rounded and epoch values can yield differences of more or less than 0.5 minutes.

### Statistical Analyses

Data from the SSM were tabulated day by day, from which weekly means and SDs were calculated. Sleep variables were analyzed using maximum likelihood estimation in a nonlinear mixed-effects model called PROC NL Mixed (SAS, version 9.4; SAS Institute Inc) [31] to establish group means and within-subject SDs (as an index of night-to-night variability), while also controlling for age and sex. A random effect on the intercept for subjects was included to account for idiosyncratic, systematic interindividual differences. Results compared the chronic insomnia group to the good-sleeper controls by means of planned contrasts determined prior to data analysis. No imputation of missing data was performed for any of the analyses, as they were not contingent upon having complete datasets. In addition, 95% CIs for fixed effects and derived contrasts were computed from the model-estimated SEs. Data are plotted for female participants (who predominated in the sample) and the grand average age (36.1, SD 12.14 years).

## Results

### Study Participants

**Figure 1.**
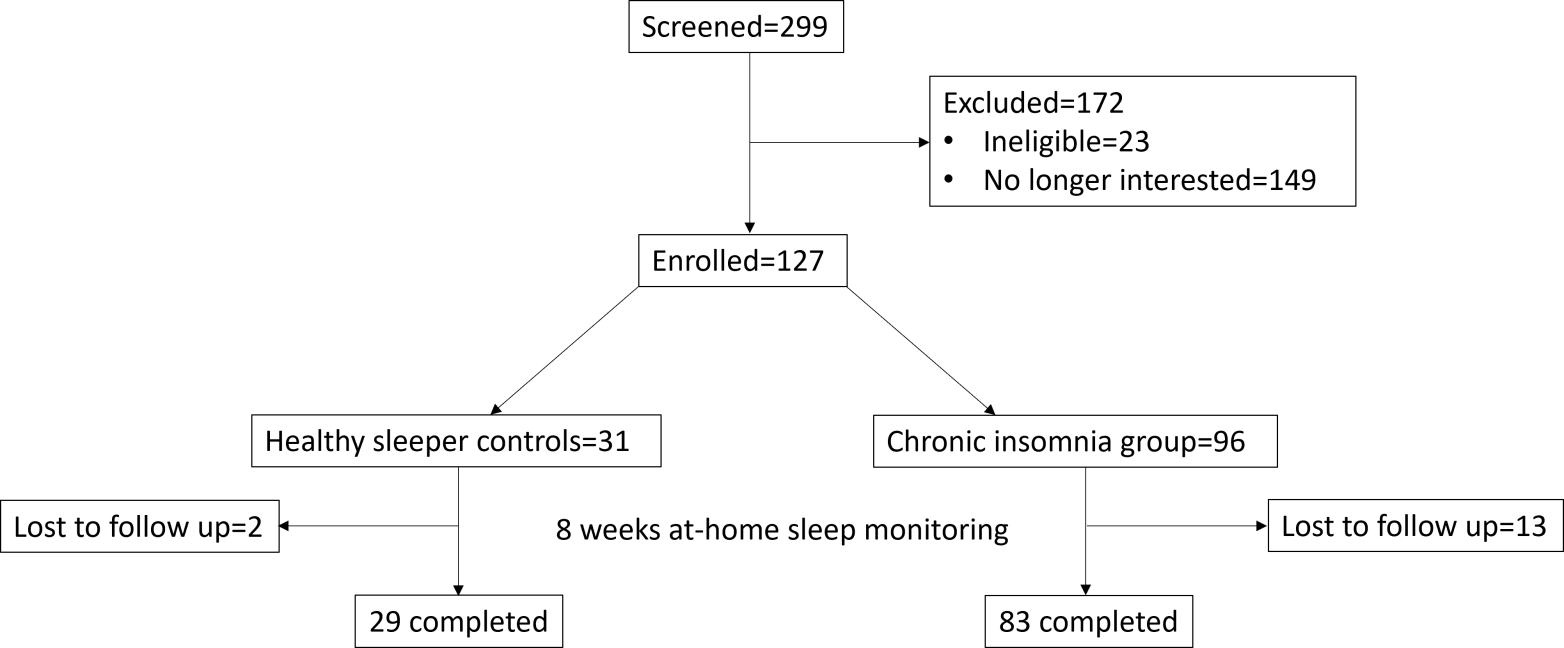
CONSORT (Consolidated Standards of Reporting Trials)–style flow diagram showing recruitment, eligibility, enrollment, group allocation, and study completion.

As shown in the CONSORT (Consolidated Standards of Reporting Trials) diagram (Figure 1), 127 participants were enrolled in the study: (n=96, 76%) adults with chronic insomnia and (n=31, 24%) healthy-sleeper controls; 112 completed the study. Table 1 shows that the final sample included 83 (74%) adults with chronic insomnia, aged 38.3 (SD 12.0) years, of which 58 (69.9%) were female. The average duration of insomnia was 8.5 years, and insomnia was of moderate severity as measured by the Insomnia Severity Index [31]. The controls were 29 (26%) healthy adults with self-reported good sleep, aged 29.7 (SD 8.8) years, of which 21 (72.4%) were female. The composition of our sample is consistent with higher prevalence rates of insomnia observed in women [32,33]. Baseline characteristics of the sample show that the insomnia group had significantly higher levels of subjective daytime sleepiness, as measured by the Epworth Sleepiness Scale [34], and sleep disruption, as measured by the Pittsburgh Sleep Quality Index [30]. They also endorsed significantly higher levels of anxiety and depression symptoms, as measured by the Generalized Anxiety Disorder 7-item scale [35] and the Patient Health Questionnaire–9 [36]. SSM data capture was generally high throughout, with an initial 97.3% (n=123) participation rate, a 91.7% (n=116) average weekly participation rate, with 83.0% (n=105) of participants using their device at least 2 nights per week across each of the 8 weeks of data collection. Across the entire 8-week period, overall use was higher in the insomnia group (75.9%) than in the healthy controls (71.8%). Overall weekly use in the insomnia group was more than 80% in weeks 1 to 2 before peaking in week 3 (83.8%), and then lowest at week 8 (63.2%). For the control group, overall use was highest in week 1 (80.8%) and lowest 69.5% in week 8 (69.5%).

**Table 1.** Baseline participant characteristics by group (N=112)^a^.

Characteristics	Insomnia group	Good-sleeper control group
Participants, n (%)	83 (74)	29 (26)
Age (years), mean (SD)	38.3 (12.0)	29.7 (8.8)
Sex, n (%)		
Female	58 (69.9)	21 (72.4)
Bedtime (hours:minutes), mean (SD)		
Workdays	23:43 (1:46)^b^	22:30 (1:17)
Days off	00:03 (1:46)^b^	22:40 (3:28)
Insomnia duration (years), mean (SD)	8.5 (9.7)	^c^—
Wake time (hours:minutes), mean (SD)		
Workdays	08:08 (2:50)	07:08 (1:11)
Days off	08:20 (2:14)	07:37 (1:28)
Assessment, mean (SD)		
Epworth Sleepiness Scale	6.3 (4.3)^d^	3.7 (3.2)
General Anxiety Disorder 7	7.1 (5.1)^e^	2.9 (3.1)
Patient Health Questionnaire–9	7.8 (4.9)^e^	1.8 (2.5)
Pittsburg Sleep Quality Index	10.7 (3.2)^e^	3.3 (2.4)
Insomnia Severity Index	17.4 (5.8)	N/A

a Group differences were assessed by independent-sample *t* test.

b *P*<.05.

c Not applicable.

d *P*<.01.

e *P*<.001.

### Characteristics of Chronic Insomnia

**Figure 2.**
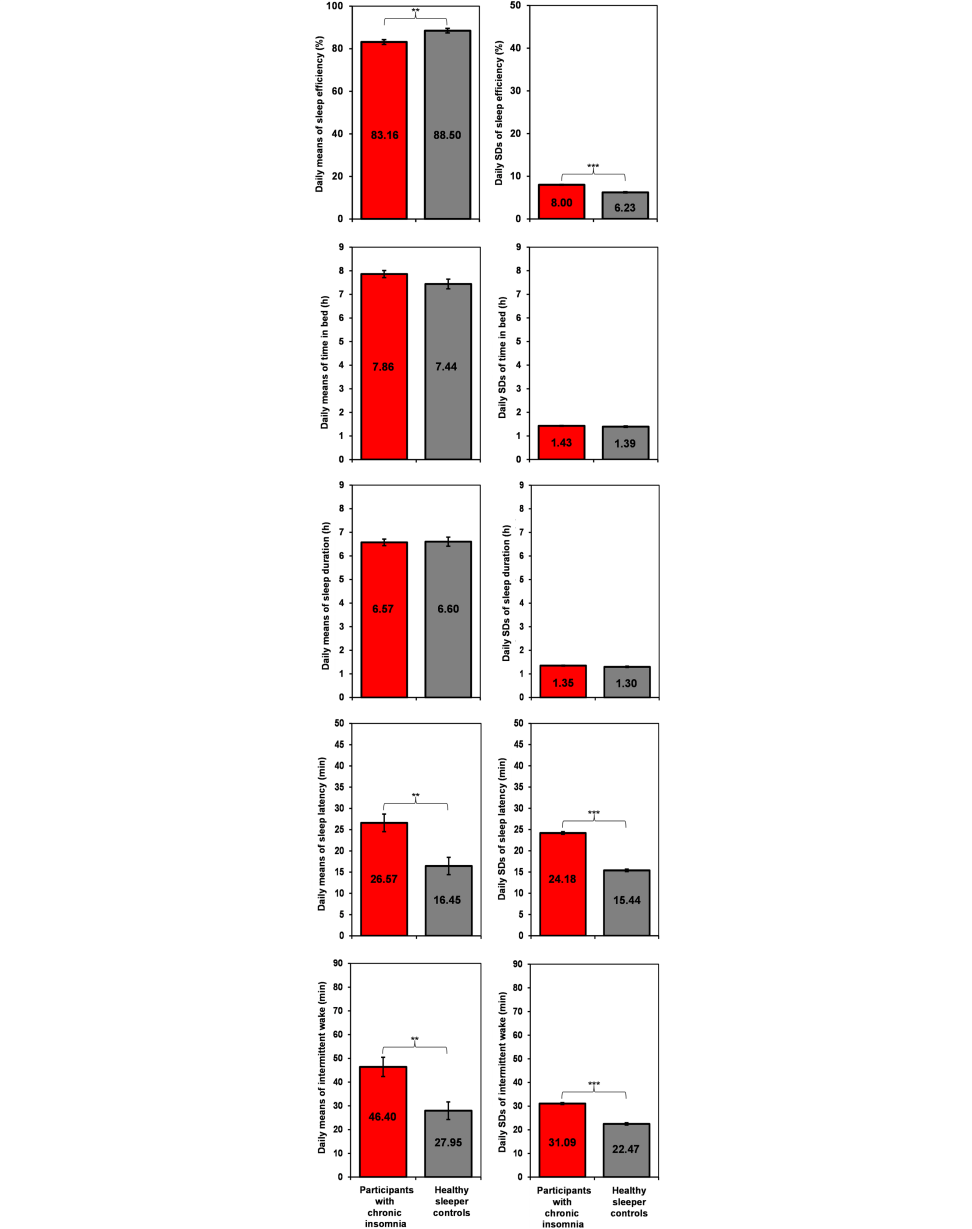
Head-to-head comparisons of sleep variables in individuals with chronic insomnia (red) vs healthy-sleeper controls (gray) for daily means (left side) and daily SDs (right side), as measured with the contactless sleep monitoring device, collapsed across days. Error bars denote SEs. **P<.01, ***P<.001.

Figure 2 shows the daily measurements made with the SSM, averaged over the duration of the study (weeks 1-8) for participants with chronic insomnia as compared to the good sleepers. Table 2 shows the comparisons between study conditions for means and SDs. Compared to the good-sleeper controls, the insomnia group had lower mean sleep efficiency (*P*=.001) with higher night-to-night variability (*P*<.001) in SDs. For sleep latency, participants with insomnia had a higher mean sleep latency (*P*=.001) and greater night-to-night variability in SDs (*P*<.001) compared to good-sleeper controls. Additionally, participants with insomnia exhibited more intermittent wakefulness both in terms of daily means (*P*=.001) and SDs (*P*<.001) as compared to the good-sleeper controls. Across groups, no significant differences were observed in mean time in bed, which averaged 7.65 (SD 0.30) hours per night (*P*=.11), or in night-to-night variability for SDs (*P*=.34). The groups also did not differ in mean sleep duration, which averaged 6.59 (SD 0.16) hours per night (*P*=.91), or in night-to-night variability for SDs (*P*=.12).

**Table 2.** Group comparison of daily means and SDs between individuals with insomnia vs healthy sleeper controls.

Participants with insomnia vs healthy sleeper controls	Sleep efficiency		Time in bed		Sleep duration		Sleep latency		Intermittent wakefulness	
	*F *(*df*)	*P* value	*F (df)*	*P* value	*F (df)*	*P* value	*F (df)*	*P* value	*F (df)*	*P* value
Means	11.45 (1, 107)	.001	2.68 (1, 107)	.11	0.01 (1, 107)	.91	12.27 (1, 107)	.001	11.54 (1, 107)	.001
SD	117.91 (1, 107)	<.001	0.91 (1, 107)	.34	2.52 (1, 107)	.12	398.08 (1, 107)	<.001	204.46 (1, 107)	<.001

To provide further context, on average, sleep efficiency was 5.34% lower, sleep latency was 10.15 minutes longer, and intermittent wakefulness was 18.45 minutes higher in the individuals with chronic insomnia than in the good-sleeper controls. Night-to-night variability was greater for sleep efficiency by 1.77%, for sleep latency by 8.80 minutes, and for intermittent wakefulness by 8.60 minutes among individuals with insomnia compared to good-sleeper controls.

## Discussion

### Principal Results

In this longitudinal naturalistic study, we observed that the SSM detected objective differences in the sleep of individuals with primary chronic insomnia compared to healthy, good-sleeper controls. Even though average time in bed and sleep duration were similar, individuals with chronic insomnia demonstrated significantly less sleep efficiency, longer sleep latency, and more intermittent wakefulness than the good-sleeper controls. Importantly, we report a novel longitudinal, objective finding of significantly greater night-to-night variability in sleep efficiency, sleep latency, and intermittent wakefulness in participants with chronic insomnia compared with good-sleeper controls.

An increased focus on night-to-night variability in studies of insomnia has become prevalent in recent literature [5,13,17-19,37,38], but technological and methodological challenges—such as smaller sample sizes [5,13,17], lack of objective measures [37], shorter observation periods [5,13,17,19,37], and a focus on older adult populations [5,17,18]—have hampered efforts to consistently demonstrate night-to-night variability. While work by Buysse et al [5] has shown greater variability in intermittent wakefulness and sleep efficiency using 2 weeks of actigraphy, this was observed in a group of older adults (mean age 71.4 years) with insomnia, and they did not find significant differences in variability in sleep latency or in mean values of any sleep measure, as found in this study.

Previous work by Wohlgemuth et al [39] argued that 1 week of recordings with polysomnography or sleep logs was sufficient to assess temporal stability in sleep parameters in elderly adults with insomnia but noted that up to 3 weeks of recordings were needed to demonstrate stability in metrics such as WASO and sleep latency. This may explain why previous research has been unable to detect the differences observed in this study and provides support for longer observational periods across wider age ranges.

We show that a validated, contactless, longitudinal CST can detect differences in sleep patterns between individuals with insomnia and healthy good-sleeper controls. Thus, the SSM can be considered a valid method to accurately assess the sleep of those with insomnia and, as such, has the potential for clinical utility in the screening of this condition and may be considered for use in insomnia medication clinical trials.

Using a noncontact, CST-enabled, automated, relatively unobtrusive recording of sleep over extended periods (8 consecutive weeks). We found evidence of substantially increased night-to-night variability in measures of sleep onset and sleep continuity that has not previously been observed. The heightened variability from day to day in these sleep characteristics and the resulting uncertainty in the sleep experience of people with chronic insomnia may contribute to the characteristic concerns and burden of this disorder. We posit that night-to-night variability is a hallmark of the chronic insomnia phenotype and that objectively and longitudinally capturing this variability in an ecologically valid manner is key to investigating and understanding the disorder.

### Limitations

This study focuses on the characterization of night-to-night variability through the examination of overall means and SDs in those with insomnia vs good-sleeper controls. The SSM also has the capability to perform sleep staging; however, as this was not the focus of this study, we are unable to comment on its accuracy.

Six of the 112 study participants completed data collection prior to the COVID-19 pandemic, and 106 were studied during the pandemic. We do not know to what degree the pandemic may have contributed to symptomology in our sample of individuals with chronic insomnia, and whether and how the pandemic may have shaped the observations in this study. Although the differences we found between individuals with chronic insomnia and good-sleeper controls are likely to be robust qualitatively, they may not generalize quantitatively to postpandemic circumstances. It is worth noting that sleep duration for our study participants averaged about 30 minutes less than the consensus recommendation of 7 or more hours per night on a regular basis [40], regardless of group or condition. However, the average sleep duration of our predominantly female sample was consistent with wearable-based findings for average sleep duration in US adults before the COVID-19 pandemic [41]. Our statistical analysis controlled for 2 specific factors (ie, age and sex) and combined all participants with insomnia into a single group. Future studies should consider including additional factors that may influence sleep patterns, as well as examining the potential effects of group assignment.

Our sample included 83 individuals with chronic insomnia and 29 healthy good-sleeper controls. While our included age range was broad (18 to 65 years), participants in our sample were relatively young overall (mean age 36.1, SD 12.1 years), which may indicate selection bias related to online recruitment efforts as well as the study requirement to have a smartphone (iPhone). Combined, these factors may have hindered access to a more representative age sample as well as those that were less economically affluent. Although the disparity in group size and age might have biased our results, it is more likely that this would have tempered, as opposed to enhanced, the observed differences between individuals with chronic insomnia and good-sleeper controls.

### Conclusions

Capturing objective, longitudinal, contactless, within-person variability of sleep and mean differences in common measures of insomnia in the home setting adds an important dimension to our understanding of poor sleep and provides a more comprehensive, ecologically valid characterization of sleep problems as experienced in daily life. This study demonstrates the potential clinical and research utility of CSTs in the characterization and potential management of insomnia and our understanding of the disease.

In this 2-month, naturalistic sleep monitoring study, we documented the novel finding of persistently elevated night-to-night variability in measures of sleep onset and sleep continuity in individuals with chronic insomnia compared to healthy good-sleeper controls. This important finding was made possible by the use of a CST device, which enabled long-term, objective, naturalistic sleep recordings in a large sample at a relatively low financial and logistical cost. Night-to-night variability may be a critical aspect of the chronic insomnia phenotype, and long-term naturalistic sleep monitoring, as demonstrated in this study, is essential to reliably document this phenomenon.

It remains to be determined whether recognition of night-to-night sleep variability as a hallmark of chronic insomnia can shed new light on some enigmatic features of the disorder, such as the discrepancy between subjective and objective daytime impairments [11,42], the heightened sensitivity to performance impairment during sleep deprivation [43], or the dampened responsiveness to stressors [44,45]. In this context, the use of contactless CST for long-term naturalistic sleep monitoring in insomnia may constitute an important methodological advancement in both research and clinical settings.

## Abbreviations

CONSORT: Consolidated Standards of Reporting Trials
CST: consumer sleep technology
OSA: obstructive sleep apnea
SSM: SleepScore Max
STOP-BANG: snoring, tiredness, observed apnea, pressure, BMI, age, neck circumference, and gender
TST: total sleep time
WASO: wake after sleep onset
